# *Prunella vulgaris* polyphenols mediate the gut-liver axis to improve MASLD: regulating cholesterol metabolism and gut microbiota

**DOI:** 10.3389/fmicb.2026.1838289

**Published:** 2026-06-17

**Authors:** Chengfeng Li, Ying Liu, Sitong Ye, Haihua Zhang, Miao Sun, Yingyan Liao, Yan Lin, Sha Tian, Kun Luo, Zhimin Zhang

**Affiliations:** 1 College of Xiangxing, Hunan University of Chinese Medicine, Changsha, China; 2 College of Pharmacy, Hunan University of Chinese Medicine, Changsha, China; 3 Key Laboratory for Quality Evaluation of Bulk Herbs of Hunan Province, Hunan University of Chinese Medicine, Changsha, China; 4 College of Integrated Chinese and Western Medicine, Hunan University of Chinese Medicine, Changsha, China

**Keywords:** bile acid, cholesterol, gut microbiota, metabolic dysfunction-associated steatotic liver disease, polyphenol

## Abstract

**Background:**

Metabolic dysfunction-associated steatotic liver disease (MASLD) is the most prevalent chronic liver disease worldwide. The latest research shows that its pathogenesis is closely related to the imbalance of gut microbiota. *Prunella vulgaris* L. is an edible-medicinal plant containing bioactive compounds such as polyphenols that can lower cholesterol and protect the liver. However, whether it has anti MASLD effects has not been reported. The present study aimed to investigate the effect of *Prunella vulgaris* polyphenols (PVP) on alleviating MASLD from the perspective of the gut-liver axis.

**Methods:**

PVP composition was characterized via UPLC-MS/MS and HPLC. Enzymatic kinetics, fluorescence quenching, and molecular docking were used to study the inhibition of PVP and rosmarinic acid (RA) on cholesterol esterase (CEase). Effects on liver lipid accumulation and intestinal cholesterol transport were assessed using HepG2 and Caco-2 cell models. A MASLD mouse model was evaluated through ELISA, tissue staining, and 16S rRNA sequencing to determine the efficacy and mechanisms.

**Results:**

PVP and RA exhibited anti-competitive inhibition of CEase, with IC_50_ values of 1.63 ± 0.06 and 0.39 ± 0.17 mg/mL, respectively. RA showed strong binding to CEase. PVP and RA significantly alleviated hepatic lipid accumulation in HepG2 cells, inhibited intestinal cholesterol absorption, and promoted cholesterol efflux in Caco-2 monolayers. In MASLD mice, PVP significantly reduced serum ALT, TBA, and TG, lowered hepatic TC and fecal TBA (*P* < 0.05), and ameliorated liver pathological damage. In addition, both PVP and RA modified the composition of gut microbiota in the cecum, which characterized by a reduction in bile acid (BA)-related bacteria such as *g_UBA7173, g_Bacteroides_H, f_Burkholderiaceae_A, g_Phocaeicola_A*, and *g_Turicimonas*, while increasing f*_Lachnospiraceae* and *f_Oscillospiraceae*.

**Conclusion:**

PVP ameliorates MASLD by inhibiting CEase and intestinal cholesterol absorption, promoting cholesterol efflux, and regulating TBA levels along with intestinal microbiota homeostasis. Our findings suggest that PVP and RA deserve further investigation as potential modulators of cholesterol metabolism in MASLD.

## Introduction

MASLD is a chronic liver disease characterized by excessive fat deposition in liver cells and metabolism related factors, after excluding alcohol and identifying liver damaging factors (Zhou et al., 2025). MASLD can gradually progress from simple hepatic steatosis to non-alcoholic steatohepatitis (NASH), leading to liver fibrosis, cirrhosis, hepatocellular carcinoma, and ultimately liver failure (Khare et al., 2025). Currently, 30.2% of the global population is affected by MASLD (Amini-Salehi et al., 2024), which poses a serious threat to human health and imposes a heavy burden on society and the economy. At present, no MASLD drug has been approved by authoritative institutions (Sutti and Albano, 2020), and the only treatment recommendation is to change lifestyle (Khare et al., 2025), which is closely related to its complex pathogenesis—the “multiple strikes” theory (Xi et al., 2025), including genetic and epigenetic factors, insulin resistance, lipid metabolism disorders, mitochondrial dysfunction and oxidative stress, endoplasmic reticulum stress, inflammatory mediators, hormones secreted by adipose tissue, and gut microbiota.

*Prunella vulgaris* L., a plant in the family Lamiaceae, is a dry fruit-spike with a bitter and pungent taste and a cold nature. Due to its edibility, *Prunella vulgaris* L. is often made into herbal tea drinks in summer, which have the effect of clearing heat and relieving summer heat, and are deeply loved by people in Asia (Bi et al., 2025). Meanwhile, it belongs to the liver and gallbladder meridians and has the effects of clearing the liver, improving vision, dispersing nodules, and reducing swelling (Chinese Pharmacopoeia Committee, 2025). Modern pharmacological research has shown that *Prunella vulgaris* L. can improve alcoholic liver disease (Rao et al., 2024), treat hepatocellular carcinoma (Lin et al., 2025), and other diseases. Terpenes, phenolic acids, flavonoids, polysaccharides, long-chain fatty acids, and vitamins are the main components of *Prunella vulgaris* L. (Lin et al., 2025), among which *Prunella vulgaris* L. polysaccharides have been shown to alleviate MASLD (Zhu et al., 2025).

Polyphenols, a class of compounds extracted from plants, including polyphenolic substances or polyphenolic monomers isolated from dietary substances or traditional medicines—belong to the category of natural products. In fact, flavonoids and phenolic acids may also have the ability to intervene and treat MASLD (Ping et al., 2025; Zhao et al., 2025). Previous studies have shown that PVP can alleviate liver lipid accumulation by upregulating ABCA1 and downregulating SREBP1 protein expression (Sun et al., 2025). RA significantly improves MASLD by repairing mitochondrial damage and regulating the YAP1/TAZ-PPARγ/PGC-1α signaling pathway (Luo et al., 2021), and alleviates oleic acid-induced hepatic steatosis in HepG2 cells by regulating endoplasmic reticulum stress and autophagy (Balachander et al., 2018).

The gut-liver axis plays an important role in the occurrence and progression of MASLD (Han et al., 2023). On the one hand, bioactive substances produced by the liver, such as BAs and antibacterial molecules, can enter the intestine to regulate the growth of the microbiota. On the other hand, the metabolites of gut microbiota can affect liver function through the gut-liver axis (Long et al., 2024). Polyphenols are a group of micronutrients widely present in plant-based foods, which can improve MASLD through the liver-gut axis. The improvement involves the regulation of gut microbiota derived metabolites, gut microbiota regulation, and the role of gut derived hormone signaling (Wang et al., 2021). Baicalin is an active ingredient extracted from the traditional Chinese medicine *Scutellaria baicalensis* Georgi, which can exert therapeutic effects on MASLD by regulating FXR BA receptors and BA metabolism (Liao et al., 2017). The polyphenols of *Prunus cerasifera* Ehrhart alleviated MASLD by inhibiting liver cholesterol biosynthesis and intestinal cholesterol absorption, as well as reducing the conversion of conjugated primary BA to lipophilic BA (Ren et al., 2024). Supplementation with silymarin alone or in combination with salvianolic acid B and puerarin improved high-fat diet induced Non-alcoholic fatty liver disease (NAFLD, now termed MASLD) by regulating gut microbiota and BA metabolism (Wang et al., 2024a).

There is currently no report on whether PVP have a therapeutic effect on MASLD. Therefore, this study investigated the inhibitory effects and mechanisms of PVP and RA on cholesterol esterase (CEase) through *in vitro* inhibitory activity, enzymatic kinetics, fluorescence quenching, and molecular docking experiments. In addition, FFA induced MASLD cell models, Caco-2 monolayer cell cholesterol transport models, and high-fat choline-deficiency diet induced MASLD mouse models were established to investigate the lipid-lowering and hepatoprotective effects and mechanisms of PVP and RA, in order to provide a theoretical basis for the development of functional foods or drugs to improve MASLD.

## Materials and methods

### Materials and reagents

*Prunella vulgaris* L. was purchased from Hunan Yafei Traditional Chinese Medicine Decoction Pieces Co., Ltd., China. The high-fat and high cholesterol choline-deficiency model feed was purchased from Shuyu Biotechnology Co., Ltd. (Shanghai, China). Oil Red O staining kit and Hematoxylin Eosin (H&E) staining kit were purchased from Soleibao Technology Co., Ltd. (Beijing, China). Total cholesterol (TC) and triglyceride (TG) assay kits were purchased from Nanjing Jiancheng Bioengineering Research Institute Co., Ltd. (Nanjing, China), while the alanine aminotransferase (ALT), TBA, CCK-8 reagent kit (E-CK-A362), and BCA protein quantification assay kits were purchased from Elabscience Biotechnology Co., Ltd. (Wuhan, China). FFA inducer (KC006, sodium oleate: palmitic acid = 2:1) was purchased from Xi'an Kunchuang Technology Development Co., Ltd. (Xi'an, China). BODIPY Cholesterol (HY-125746), Lucifer Yellow CH (HY-128692), and Yizhemai Bu (HY-17376) were purchased from MCE. Improved Eagle culture medium (DMEM, PM150210), heat inactivated fetal bovine serum (FBS, 164260-50), and HBSS (PB180326) were purchased from Wuhan Punosai Life Technology Co., Ltd. (Wuhan, China). Cholesterol (C3045) was purchased from Sigma. Cell cholesterol detection kit (E1015-105) was purchased from Beijing Pulilai Gene Technology Co., Ltd. (Beijing, China). Cholesterol esterase (V34822-100U) and caffeic acid (purity ≥ 98%) were purchased from Shanghai Yuanye Biotechnology Co., Ltd. (Shanghai, China). 4-Nitrophenylbutyrate (pNPB, purity ≥ 98%) was purchased from Aladdin Biochemical Technology Co., Ltd. (China). Salviaflaside (purity ≥ 98%) was purchased from Chengdu Efa Biotechnology Co., Ltd. (Chengdu, China). RA (purity ≥ 98%) was purchased from Chengdu Ruifensidan Biotechnology Co., Ltd. (Chengdu, China). Acetonitrile, methanol and other chemical reagents are analytical grade.

### Animals and cell lines

Six-week-old SPF grade male C57BL/6J mice were purchased from Hunan Slack Jingda Experimental Animal Co., Ltd. and housed in the SPF grade animal room of the Experimental Animal Center of Hunan University of Traditional Chinese Medicine. The environmental temperature was (23 ± 2)°C, humidity was (55 ± 5)%, and the light cycle was 12/12 h.

The HepG2 cell line (CL-0103) and Caco-2 cell line (CL-0050) were both purchased from Wuhan Procell Life Science and Technology Co., Ltd. HepG2 cells were cultured in a basic medium containing 10% fetal bovine serum and 1% penicillin/streptomycin in a 37°C, 5% CO_2_ incubator. Based on the cell growth status, when the cell confluence reached around 90%, cells were collected and passaged using 0.25% trypsin digestion. Logarithmic growth stage cells were taken for experimentation.

### Preparation of PVP

The preparation of PVP followed the research method of (Luo et al. 2024), with slight modifications. *Prunella vulgaris* L. powder was weighed and extracted with 10 times the volume of 70% ethanol by refluxing for 1 h, repeating the process three times. The extract was filtered and concentrated, then transferred to an AB-8 macroporous resin chromatography column, and eluted sequentially with five times the column volume of pure water and 50% ethanol. The 50% ethanol elution fraction was collected, concentrated, and lyophilized.

### Determination of total phenolic and total flavonoid content

#### Determination of total phenolic content in PVP

The total phenolic content of PVP was determined using the Folin-Ciocalteu method (Biglari et al., 2008). 0.20 g of PVP lyophilized powder was precisely weighed, dissolved ultrasonically in 5 mL of anhydrous methanol, and then 0.2 mL of the above solution was diluted to obtain a PVP solution of 0.8 mg/mL. Nest, 0.50 mL of the PVP solution was mixed with 2 mL of deionized water and 0.50 mL of Folin-Ciocalteu reagent. Then 5 mL of 7% sodium carbonate solution and 4 mL of deionized water were added. After reacted at room temperature for 90 min, the mixture was measure at OD_760_. Gallic acid was used as a standard to generate a standard calibration curve, and the results are expressed in mg gallic acid equivalent (GAE) per gram dry weight (DW).

#### Determination of total flavonoid content in PVP

The total flavonoid content of PVP was determined using the AlCl3 colorimetric method (Biglari et al., 2008). One milliliter of PVP solution (0.8 mg/mL) was reacted with 0.70 mL of NaNO_3_ solution (50 mmol/L) for 7 min, and then 0.3 mL of AlCl3 solution (100 mg/mL) was added. After 6 min of reaction, the mixture was added with 5.0 mL of 1 mol/L NaOH solution in the dark at room temperature for 15 min, then centrifuged. The supernatant was measured at OD_510_. Rutin was used as a standard to generate a standard calibration curve, and the results are expressed as mg rutin per gram dry weight (DW).

#### UPLC-MS/MS component identification

Refer to Lin et al.'s method (Lin et al., 2021) for component identification of PVP and make slight modifications. Firstly, 100 mg of PVP freeze-dried powder was mixed with 500 μL of pre cooled extraction solvent (methanol: water = 4:1, including internal standard 1,000:10) and two small steel balls at −40°C. After homogenized at 45 Hz for 4 min, sonicated in an ice water bath for 1 h, and maintained at 40°C for 1 h, the extract was centrifuged at 4°C and 12,000 rpm for 15 min. After filtration through a 0.22 μM microporous membrane, 100 μL of supernatant was taken for sample analysis. According to the reference, LC-MS/MS analysis was performed on an UHPLC system (Vanquish, Thermo Fisher Scientific) coupled with an ACQUITY UPLC^®^ HSS T3 column (1.8 μm, 2.1 × 100 mm; Waters, USA) and Thermo Q Exactive mass spectrometer (Thermo Fisher Scientific, USA). MS and MS/MS data were obtained in IDA acquisition mode.

### HPLC quantitative analysis of PVP

#### HPLC content determination

Twenty milligrams of PVP freeze-dried powder was accurately weighed to prepare PVP test solution (2 mg/mL) with methanol. 4.80, 1.04, and 3.70 mg of caffeic acid, salviaflaside, and RA were accurately weighed to prepare standard reference solution. Appropriate amounts of standard reference solution was took separately, diluted with methanol to form a mixed standard solution, and filtered through a 0.2 μm polyvinylidene fluoride (PVDF) syringe filter. The Agilent 1260 high-performance liquid chromatography system (Agilent) was used to characterize the composition of total phenols in *Prunella vulgaris*. The sample was separated at 30°C using a chromatographic column of Elite Supersil ODS2 (4.6 mm × 250 mm, 5 μm). The mobile phase consists of acetonitrile (A) −0.1% acetic acid solution (B), and the elution gradient is set as follows: 10%−12% A (0–6 min), 12%−15% A (6–16 min), 15%−19%A (16–18 min), 19%−21%A (18–28 min), 21%−26%A (28–29 min), 26%−29%A (29–35 min), 29%−45%A (35–40 min), 45%−10%A (40–45 min). The sample (10 μL) was injected at a flow rate of 1.0 mL/min, with detection wavelength of 330 nm.

#### Methodological investigation

Linear relationship investigation: 4.80 mg of caffeic acid, 1.04 mg of salviaflaside, and 3.70 mg of RA were precisely weighed to prepare caffeic acid standard solution (0.0096, 0.0192, 0.0384, 0.0768, 0.096, and 0.1157 mg/mL), salviaflaside standard solution (0.00832, 0.01664, 0.0208, 0.0251, 0.0832, and 0.2080 mg/mL), and RA standard solution (0.0148, 0.0296, 0.0592, 0.0740, 0.1480, and 0.2960 mg/mL), using methanol as the solvent. Ten microliters of the above standard solution was injected into the liquid chromatograph, with the injection concentration (*X*, mg/mL) as the *x*-axis and peak area (*Y*) as the *y*-axis to obtain the regression equation.

Precision: Take the same sample and inject it continuously for six times. If the relative retention time RSD of each spectral peak is less than 1% and the relative peak area RSD is less than 2%, it indicates that the instrument has good precision.

Stability: Take the same sample solution and inject 10 μL sequentially at 0, 2, 4, 8, 12, and 24 h. The relative peak area RSD of each color spectrum peak is less than 3%, indicating that the sample is stable within 24 h.

Repeatability: The test solution of six samples was prepared and detected, and the RSD of each component content is less than 3%, indicating good repeatability.

Recovery rate of sample addition: caffeic acid, salviaflaside, and RA were mixed into six samples with known contents to detect.

### Inhibition of CEase and kinetics

#### The inhibitory effect of PVP on CEase

The study on *in vitro* inhibition of CEase activity referred to Long et al.'s method with slight modifications (Long et al., 2022). Fifteen microliters of CEase solution (0.96 U/mL) was mixed with 50 μL of PVP solution (0, 0.3, 0.6, 0.9, 1.2, 1.5, 1.8, 2.1, and 2.4 mg/mL) or RA solution (0, 0.03, 0.06, 0.09, 0.12, 0.15, 0.18, 0.21, and 0.24 mg/mL) of different concentrations, followed by 1,000 μL of PBS buffer (pH 7.4). After incubating the mixture at 25°C for 10 min, 10 μL of substrate pNPB (1 mg/mL) was added. Immediately, the absorbance value was measured using a TU1900 UV visible spectrophotometer [Beijing Spectral Analysis General Instrument Co., Ltd. (China)] at 405 nm. The system without enzymes was used as the experimental blank group, the system without inhibitors was used as the control group, and the system without enzymes and inhibitors was used as the control blank group. Repeat the experiment three times and calculate the inhibitory effect of PVP or RA on CEase using Equation 1.


$$\begin{array}{l} Inhibition\left(\%\right)=\frac{A-B-\left(C-D\right)}{A-B}\times 100\% \end{array}$$ (1)

Among them, *A* and *B* are the absorbance of blank controls with or without enzymes, respectively. *C* and *D* are the absorbance of PVP or RA with or without enzymes, respectively.

#### Inhibition kinetics of PVP on CEase

Determination of inhibition mode of PVP or RA on CEase (Shen et al., 2023): 1 mg/mL pNPB, different concentrations of PVP solution (0, 0.45, and 0.9 mg/mL) or RA solution (0, 0.045, and 0.09 mg/mL), and different concentrations of CEase (0.15, 0.3, 0.45, 0.6, and 0.75 U/mL) were reacted for 5 min, and the changes in absorbance values were measured. Graphpad software was used to draw a curve of enzymatic reaction rate (ΔOD/min) vs. CEase concentration, and the inhibitory effect of the compound on CEase was determined based on the characteristics of the curve.

Determination of inhibition types of PVP on CEase (Shen et al., 2023): Different concentrations of PVP solution (0, 0.45, and 0.9 mg/mL) or RA solution (0, 0.045, and 0.09 mg/mL), and different concentrations of pNPB solution (0.5, 1, 1.5, 2.0, and 2.5 mg/mL) were reacted for 30 min with the concentration of CEase fixed at 2.5 mg/mL. The changes in absorbance values was measured. A double reciprocal plot with the reciprocal 1/[S] of pNPB concentration as the horizontal axis and the reciprocal 1/*v* of enzymatic reaction rate (ΔOD/min) as the vertical axis was plotted by Graphpad software. Determine the type of inhibition based on the characteristics of the plot and enzyme kinetic parameters such as *K*_*m*_ and *V*_*max*_.

#### Fluorescence quenching measurements

According to Ji et al.'s method (Ji et al., 2021), the Cary Eclips fluorescence spectrometer (Agilent; ; BioTek Instruments, Inc., USA) was used to determine the effect of different concentrations of PVP or RA solutions on the fluorescence spectra of CEase. 1.5 mL of CEase solution (0.24 mg/mL) was mixed with 50 μL of PVP solution (0, 0.92, 1.83, 2.75, and 3.67 mg/mL) or 50 μL of RA solution (0, 0.092, 0.183, 0.275, and 0.367 mg/mL) for 1 min and incubated at 37°C for 1 h. After diluted with phosphate buffer solution, the mixture was used to measure the effect of PVP or RA on CEase fluorescence spectrum by Cary Eclips fluorescence spectrometer. The fluorescence spectral parameters are as follows: excitation wavelength of 280 nm, emission wavelength of 300–500 nm, slit width of the excitation band of 5 nm, slit width of the emission light of 10 nm, data interval of −1 nm, scanning speed of Medium. Fluorescence quenching was determined using Stern Volmer equation (Equation 2):


$$\begin{array}{l} \frac{F_{0}}{F}=\text{1}+Ksv\;\left[Q\right] \end{array}$$ (2)

Among them, *F*_0_ is the fluorescence intensity without PVP, and *F* is the fluorescence intensity after adding PVP. *Ksv* is the Stern Volmer quenching constant, and [*Q*] is the concentration of PVP (mg/mL).

The number of binding sites (*n*) and the binding constant (*K*α) are obtained through equation (Equation 3):


$$\begin{array}{l} \text{Lg}\frac{\left(F_{0}-F\right)}{F}\text{=}\mathrm{lg}K\alpha +n\text{lg}\left[Q\right] \end{array}$$ (3)

### Establishment of MASLD cell model and PVP intervention

#### Cell viability

Referring to the experiment of Li et al. (2024a), cell viability of HepG2 cells was assessed using the CCK-8 assay kit. An experimental group, a control group, and a blank group were set up with six wells in each group. HepG2 cells in logarithmic growth phase were seeded onto 96 well-plates at a density of 1 × 10^4^/well and cultured in DMEM medium containing 10% fetal bovine serum, 1% penicillin/streptomycin, and 4.5 g/L glucose. The cells were then incubated in a cell culture incubator at 37°C with 5% CO_2_. After incubating for 24 h, the culture medium was removed. The experimental groups were supplemented with 100 μL of drug-containing medium (with PVP concentrations of 25, 50, 100, 200, 400, 800, and 1,000 μg/mL), while the control and blank groups were supplemented with 100 μL of culture medium. After continuing the culture for another 24 h, 10 μL of CCK-8 solution was added to each well, followed by further incubation in the incubator for 2 h. The absorbance was then measured at 450 nm using a microplate reader. Cell viability was calculated according to Equation 4:


$$\begin{array}{l} Cell\;viability\%=\left[\left(A_{1}-A_{0}\right)/\;\left(A_{2}-A_{0}\right)\right]\times 100\% \end{array}$$ (4)

Among them, *A*_1_ is the absorbance value of the experimental well, *A*_2_ is the absorbance value of the control well, and *A*_0_ is the absorbance value of the blank well.

#### Oil Red O staining

FFA induced lipid accumulation in HepG2 cells has been established as a cell model for MASLD, following the experimental protocol of Pan et al. (2023), with slight modifications. HepG2 cells were treated with a free fatty acid (FFA) inducer (12 mM sodium oleate: 6 mM palmitic acid) to establish a MASLD model.

HepG2 cells in logarithmic growth phase were seeded onto 24 well-plates with a cell density of 1 × 10^6^ per well. They were cultured in a non-fatty acid DMEM medium containing 10% fetal bovine serum, 1% penicillin/streptomycin, and 4.5 g/L glucose. The cells were then incubated in a cell culture box containing 5% CO_2_ at 37°C for 24 h until they fully adhered to the cell wall. After suctioning off the culture medium, proceed as follows:

Blank group: 400 μL non-fatty acid DMEM medium; Model group: 400 μL of fatty acid free DMEM medium containing 0.20 mmol/L FFA; Positive control group: 400 μL of non-fatty acid DMEM medium containing 5 μmol/L lovastatin and 0.20 mmol/L FFA; Experimental group: 400 μL of fatty acid free DMEM medium containing low and high concentrations of PVP (50, 100 μg/mL) and 0.2 mmol/L FFA, respectively. Each sample has four replicates.

After 24 h of incubation, the cells were discarded from the culture medium and rinsed three times with PBS. According to the instructions of the Oil Red O staining kit, lipid droplets and cell nuclei were stained, and the formation of red lipid droplets inside the cells was observed and photographed under an Axiocam 208 color microscope (Zeiss; 200 × field).

### *In vitro* test in cholesterol transport model

#### Cell culture

Caco-2 cells were seeded in culture bottles at 37°C with 5% CO_2_. The culture medium is DMEM high glucose medium containing 10% FBS, 1% penicillin streptomycin, 2 mM L-glutamic acid, and 1% non-essential amino acids. Passage was carried out when the cell density reached 80%−90%.

#### Cell viability

Caco-2 cells in logarithmic growth phase were seeded in 96 well-plates with 1 × 10^4^ cells per well and cultured at 37°C with 5% CO_2_ until they adhered to the wall. Then, the cells were divided into eight PVP groups (6.25, 12.5, 25, 50, 100, 200, 400, and 800 μg/mL), eight RA groups (6.25, 12.5, 25, 50, 100, 200, 400, and 800 μg/mL), and a control group (containing only cells, without drug), with three replicates in each group. After 24 h of drug action, 20 μL of CCK-8 solution (5 mg/mL) was added to each well in the dark and incubated for 4 h. Then, the supernatant was absorbed and 150 μL of dimethyl sulfoxide was added to each well. After shaking for 10 min, the OD values of each well were measured at 490 nm using a BIOTEK/Synergy multifunctional measuring instrument (BioTek Instruments, Inc.). Cell viability was calculated according to Equation 4.

#### Cell transmembrane resistance detection (TEER measurement)

Caco-2 cells were seeded into six-well TransWeLL^TM^ plates at a rate of 1 × 10^5^ cells/cm^2^ (approximately 3 × 10^5^ cells/mL), with 1.5 mL of culture medium in the upper chamber and 2.6 mL of culture medium in the lower chamber (Tayyeb et al., 2021), and cultured at 37°C with 5% CO_2_. The culture medium is DMEM containing 10% FBS, 1% penicillin streptomycin, 2 mM L-glutamate, and 1% non-essential amino acids. The cell membrane resistance was measured on Day 7, Day 14, and Day 21, respectively (de Souza Figueira et al., 2022). TEER value was calculated according to Equation 5:


$$\begin{array}{l} TEER=\left(R-R_{0}\right)\times S \end{array}$$ (5)

Among them, *R* is the transmembrane resistance value of the experimental group, *R*_0_ is the transmembrane resistance value of the blank group, and *S* is the surface area of the cell monolayer, which is the effective membrane area of the upper chamber of TransWeLL^TM^ (cm^2^).

#### Lucifer Yellow CH transport experiment

Lucifer Yellow CH standard curve: Standard solutions were prepared with concentrations of 0.015, 0.02, 0.04, 0.06, and 0.08 mg/mL, and measured the fluorescence intensity. Then, a standard curve was plotted with Lucifer Yellow CH concentration as the *x*-axis and fluorescence intensity as the *y*-axis. The measured standard curve is *Y* = 0.0161*X*−0.0008, and *R*^2^=0.9987.

The six-well TransWeLL^TM^ plate of Caco-2 cells (TEER value ≥ 600 Ω × cm^2^) that have been cultured for 21 days and reached the plateau stage of resistance value was performed for Lucifer Yellow CH transport experiment (Guo et al., 2024). Firstly, after removed the culture medium, the plate was washed three times with HBSS preheated to 37°C on the AP side and BL side, respectively, and added HBSS buffer for the last time. After equilibration at 37°C for 20–30 min, 1.5 mL of fluorescent yellow (100 μM, HBSS preparation, 37°C) was added to the AP side and 2.6 mL of HBSS (37°C) was added to the BL side, and incubated in a 37°C incubator for 150 min. At 30, 60, 90, 120, and 150 min, 100 μL of samples were taken from the BL side, and 100 μL of HBSS was added. Fluorescence intensity was measured using BIOTEK/Synergy multifunctional measuring instrument (BioTek Instruments, Inc.) at an excitation wavelength of 428 nm and an emission wavelength of 536 nm on the BL side.

According to the standard curve, LY concentration in the BL side transport solution was calculated. Then, the apparent permeability coefficient (Papp) can calculate according to Equation 6.


$$\begin{array}{l} Papp=\Delta Q/\left(\Delta t\times A\times C_{0}\right) \end{array}$$ (6)

Δ*Q* is the transmittance of fluorescent yellow during the Δ*t* time period, *A* is the surface area of the cell monolayer, that is, the effective membrane area of the upper chamber of TransWeLL^TM^ (cm^2^), and *C*_0_ is the initial concentration of fluorescent yellow on the AP side (μg/mL).

#### BODIPY-cholesterol transport experiment

HepG2 cells were seeded at a concentration of 3 × 10^5^ cells/mL in six-well-plates and cultured for 48 h (Ren et al., 2024). Then, the Caco-2 cells cultured for 21 days, with a plateau resistance value reaching the plateau phase (TEER value ≥ 600 Ω· cm^2^, forming a cell monolayer simulating intestinal epithelial barrier) and Papp < 1 × 10^−6^ cm/s were transferred to the top of six-well-plates containing 90% fused HepG2 cells at the bottom. Caco-2 and HepG2 cells were incubated together in a co culture system to reconstruct bidirectional interactions between the gut and liver (Yao et al., 2020). Grouping was conducted (CON, MOD, POS, PVP-L, PVP-M, PVP-H, RA-L, RA-M, and RA-H, with three parallel cells in each group), and 1.5 mL of different doses of drugs were added to the AP side (prepared with fresh culture medium and supplemented with DMSO as needed, but the final concentration of DMSO in the culture medium did not exceed 0.1%) to treat the cells for 24 h (2.6 mL of fresh culture medium was added to the BL side). Then, remove the TransWeLL^TM^ plate from the incubator and gently rinse the upper and lower chambers twice with HBSS preheated at 37°C. Examination of cholesterol transport from AP to BL (de Souza Figueira et al., 2022): 1.5 mL of BODIPY Cholesterol (10 μM, HBSS preparation) preheated at 37°C was added to the upper chamber (AP side). The CON group was replaced with HBSS, and 2.6 mL of HBSS (37°C) was added to the lower chamber (BL side). After 2 h, samples were taken from the lower chamber (BL side) and added to black 96 well-plates (100 μL/well). The fluorescence intensity was detected using BIOTEK/Synergy multifunctional measuring instrument (BioTek Instruments, Inc.) under excitation wavelength of 482 nm and emission wavelength of 515 nm. Examination of cholesterol transport from BL to AP (Guo et al., 2024): 1.5 mL of HBSS (37°C) was added to the upper chamber (AP side), and 2.6 mL of Cholesterol preheated at 37°C was added to the lower chamber (BL side). After 2 h, samples were taken from the upper chamber (AP side), and the cholesterol content in the samples was determined using a free cholesterol (FC) colorimetric test kit.

### Molecular docking

AutoDockTools-1.5.7 (USA) software was used for molecular docking. Firstly, the three-dimensional crystal structure of cholesterol esterase (PDB ID: 1AQL) was retrieved from the PDB. The structure was then modified by removing water molecules and impurities, followed by the addition of hydrogen atoms, and subsequently exported in pdbqt format. The 3D structure of RA was downloaded from the NCBI website and imported into AutoDock software, after which it was exported in pdbqt format. Using the “AutoDockTools” module, the binding catalytic sites of cholesterol esterase enzyme and RA was predicted. The Lamarckian Genetic Algorithm was employed, and “Run AutoDock Vina” was selected to perform precise docking simulations. The binding energies of different conformations of RA with enzymes were calculated, and the conformation with the lowest binding energy was considered to the optimal interaction, thereby determining the best conformation and binding site of RA with cholesterol esterase. Finally, PyMol software was used to generate a visualization of the optimal docking interaction.

### Animal experimentation

#### Modeling and drug intervention

After 1 week of adaptive feeding, 20 SPF grade C57BL/6J mice (6 weeks old) were randomly divided into four groups: Normal group, received a basal diet, and daily oral gavage of physiological saline; MASLD group, fed a choline-deficient high-fat diet, and administered physiological saline via oral gavage daily; PVP group, maintained on a choline-deficient high-fat diet with a daily oral gavage of 200 mg/kg PVP (dissolved in physiological saline); RA group, fed a choline-deficient high-fat diet with a daily oral gavage of 60 mg/kg RA (dissolved in physiological saline). The chosen doses for daily oral gavage is based on comprehensive consideration the normalization method of body surface area and previous study by our research group. Body weight was recorded weekly. The relevant animal experiment plan has been approved by the Animal Ethics Committee of Hunan University of Traditional Chinese Medicine (No. SLBH-202312120004).

After 8 weeks, the experimental animals were fasted for 12 h with water available, and feces samples from each mouse were collected and stored at −80°C for subsequent analysis. The mice were anesthetized via intraperitoneal injection of sodium pentobarbital. Blood was collected from the abdominal aorta and centrifuged in a low-temperature high-speed centrifuge at 3,000 r/min for 10 min. After centrifugation, the supernatant was used for later biochemical analysis. Following blood collection, the liver was removed and weighed to determine the liver index. Then, a portion of the liver tissue was fixed in 4% paraformaldehyde, while another portion was stored at −80°C. Cecum contents were also collected and stored at −80°C. The liver index was calculated using Equation 7.


$$\begin{array}{l} Liver\;coefficient=\frac{liver\;weight}{body\;weight}\times 100\% \end{array}$$ (7)

#### Biochemical indicator detection

Serum ALT, TG, and TC levels were measured according to the instructions of the respective assay kits. Liver tissue or feces samples were collected from mice, and total protein was extracted using the BCA protein assay kit. Subsequently, biochemical indicators including hepatic TG, TC, and TBA, as well as fecal TBA, were analyzed in accordance with the manufacturer's instructions.

#### Pathological examination of liver tissue

Fresh liver tissue samples were completely fixed with 4% paraformaldehyde for 48 h, followed by dehydration and clearing with xylene, and embedded in paraffin. Subsequently, the tissues were sectioned into 4 μm thick paraffin sections. After deparaffinization and hydration, the sections were stained with hematoxylin (AWI0001a, Changsha Abiowell Biotechnology Co., Ltd.)—Yihong (AWI0029a, Changsha Abiowell Biotechnology Co., Ltd.). Images were acquired using an inverted fluorescence microscope.

#### 16S rRNA sequencing analysis of cecal contents

Total DNA of gut microbiota was extracted from mouse cecal contents using the CTBA method. The variable region of the bacterial 16S rRNA gene was amplified by PCR with the primer pair5′-ACTCCTACGGGAGGCAGCA3′ and5′-GACTACHVGGGTWTCTAAT3′. Amplification was performed using a 2720 PCR Thermal Cycler (ABI Company). All samples were amplified in triplicate. Subsequently, the amplified product was purified and recovered using magnetic beads (Vazyme VAHTSTM DNA Clean Beams), and the purified product was quantified with a Microplate reader (BioTek, FLx800). Finally, Illumina's TruSeq Nano DNA LT Library Prep Kit was used to construct the sequencing library. Paired-end sequencing was performed on the Illumina NovaSeq 6000 platform. Relative abundances and gut microbiota diversity, along with intergroup differences at different taxonomic levels were analyzed using QIIME2 (https://docs.qiime2.org/2022.11/tutorials/). Alpha diversity was assessed using indices including Chao1, Observed_features, Shannon, Simpson, Faith's PD, Pielou's evenness, and Good's coverage. Beta diversity was evaluated via Principal Coordinate Analysis (PCoA) and Non Metric Multidimensional Scaling (NMDS) reflect. Differences in species distribution and identification of marker species were analyzed using PCA, LEfSe, OPLS-DA, and random forest analysis.

### Statistical analysis

Linear fitting was performed using Origin 8.5 software. All data represent at least three independent experiments. Statistical analysis was conducted with GraphPad Prism 8.0 software, using one-way ANOVA combined with Dunnet's multiple comparison test or Tukey's multiple comparison test to determine significance. The Data are expressed as mean ± standard error (SEM), and *p* < 0.05 is considered statistically significant.

## Results

### The total phenols and total flavonoids content of PVP

The extraction yield of PVP was (3.75 ± 0.325)%. The total phenolic and flavonoid contents in PVP were determined by the Folin-phenol method and the aluminum nitrate-sodium nitrite colorimetric method. As shown in Table 1, the total phenolic content was (29.00 ± 0.496)%, and the total flavonoid content was (46.93 ± 0.145)%.

**Table 1:** Extraction rate, total phenolic content, and total flavonoid content of PVP.

Extraction rate (%)	Total phenols content (%)	Total flavonoids content (%)
3.75 ± 0.325	29.00 ± 0.496	46.93 ± 0.145

### Characterization of PVP by UPLC-MS/MS

**Figure 1:**
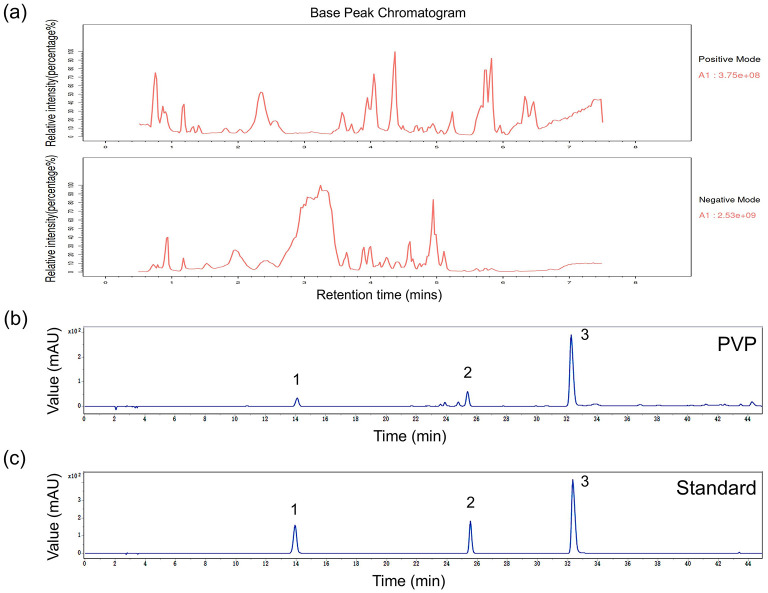
Composition characterization of PVP. **(a)** UPLC-MS/MS. HPLC chromatograms of the test sample and **(b)** the mixed reference standard **(c)**. (1) Caffeic acid, (2) Salviaflaside, and (3) RA.

The substance identification process involved searching and comparing against databases such as the Human Metabolome Database (HMDB, http://www.hmdb.ca), MassBank (https://massbank.jp/), LipidMaps (http://www.lipidmaps.org), mzClound (https://www.mzcloud.org), and KEGG (https://www.genome.jp/kegg/), combined with references. The molecular weight and adduct ion information of metabolites were determined based on the mass-to-charge ratio of precursor ions in primary mass spectrometry, enabling molecular formula prediction. These predictions were then compared and matched against the databases. Additionally, metabolites in the quantitative list with secondary mass spectra were compared and matched against fragment ion information from secondary spectra in the databases to achieve secondary qualitative identification of metabolites. The results indicated that PVP primarily consists of flavonoids and phenolic acids, including quercetin 3-rhamnoside-7-glucoside, caffeic acid, rutin, chrysosplenetin, and RA (Table 2 and Figure 1a).

**Table 2:** Main components of PVP.

Name	mz	rt/s	Formula	Precursor_type	CAS	HMDB	Classification
Oleuroside	539.1487	49.5	C_25_H_32_O_13_	[M–H]^−^	116383-31-4	HMDB0035352	Polyphenols
Pelargonidin	543.1299	51.8	[C_15_H_11_O_5_]+	[2M+H]^+^	134-04-3	HMDB0003263	Flavonoids
Galloyl glucose	377.0781	72.8	C_13_H_16_O_10_	[M+HCOO]^−^	554-37-0	HMDB0301708	Polyphenols
Hydroxyhydroquinone	127.0388	75.1	C_6_H_6_O_3_	[M+H]^+^	533-73-3	HMDB0124831	Polyphenols
27,7-Dihydroxyflavone	315.0509	96	C_15_H_10_O_4_	[M+HCO3]^−^	2196-14-7	HMDB0247290	Flavonoids
3,5-Dimethoxyphenol	153.0556	128	C_8_H_10_O_3_	[M–H]^−^	500-99-2	HMDB0059966	Phenols
Neochlorogenic acid	355.1026	186.3	C_16_H_18_O_9_	[M+H]^+^	906-33-2	HMDB0240477	Phenolic acids
Sophorol	299.0548	217.1	C_16_H_12_O_6_	[M–H]^−^	524-08-3	HMDB0301810	Flavonoids
Nevadensin	687.1794	217.1	C_18_H_16_O_7_	[2M–H]^−^	10176-66-6	HMDB0255572	Flavonoids
Chrysosplenetin	373.0923	223.3	C_19_H_18_O_8_	[M–H]^−^	603-56-5	HMDB0250217	Flavonoids
Rosmarinic acid	361.0924	225.6	C_18_H_16_O_8_	[M+H]^+^	20283-92-5	HMDB0003572	Phenolic acids
Quercetin 3-rhamnoside-7-glucoside	609.1426	234	C_27_H_30_O_16_	[M–H]^−^	17306-45-5	HMDB0135150	Flavonoids
Rutin	611.1595	237.1	C_27_H_30_O_16_	[M+H]^+^	153-18-4	HMDB0003249	Flavonoids
Quercetin 3-galactoside	927.1806	238.8	C_21_H_20_O_12_	[2M–H]^−^	482-36-0	HMDB0030775	Flavonoids
Isoquercitrin	927.194	238.8	C_21_H_20_O_12_	[2M–H]^−^	482-35-9	HMDB0037362	Flavonoids
Quercetin 3-(6′-malonyl-glucoside)	551.1029	248.3	C_24_H_22_O_15_	[M+H]^+^	96862-01-0	HMDB0037368	Flavonoids
Sagerinic acid	738.2058	251	C_36_H_32_O_16_	[M+NH4]^+^	238752-75-5	HMDB0033600	Polyphenol
Delphinidin 3-glucoside	449.1071	251	[C_21_H_21_O_12_]+	[M–OH+H]^+^	6906-38-3	HMDB0037997	Flavonoids
Quercitrin	447.0927	258	C_21_H_20_O_11_	[M–H]^−^	522-12-3	HMDB0033751	Flavonoids
4-Hydroxyphenylpyruvic acid	361.0925	262.1	C_9_H_8_O_4_	[2M+H]^+^	156-39-8	HMDB0000707	Phenols
Salicin	325.2017	276.2	C_13_H_18_O_7_	[M+K]^+^	138-52-3	HMDB0003546	Phenols
Vanillin	191.1434	276.2	C_8_H_8_O_3_	[M+K]^+^	121-33-5	HMDB0012308	Phenols
Gardenin B	357.0971	277	C_19_H_18_O_7_	[M–H]^−^	2798-20-1	HMDB0302154	Flavonoids
Kaempferol	285.04	282	C_15_H_10_O_6_	[M–H]^−^	520-18-3	HMDB0005801	Flavonoids
Casticin	373.0925	287.3	C_19_H_18_O_8_	[M–H]^−^	479-91-4	HMDB0030660	Flavonoids
Formononetin	267.0663	291.3	C_16_H_12_O_4_	[M–H]^−^	485-72-3	HMDB0005808	Flavonoids
Caffeic acid	163.0387	312.6	C_9_H_8_O_4_	[M–H2O+H]^+^	331-39-5	HMDB0001964	Phenolic acids

### HPLC analysis of PVP

The high-performance liquid chromatogram of PVP freeze-dried powder is shown in Figures 1b, c. This method has been validated in terms of linearity, precision, stability, repeatability, and recovery rate.

Linear test: The standard curve equations and linear ranges of each component are detailed in Table 3. There is a good linear correlation between the concentration (*X*) of the target compound and its peak area (*Y*) within the tested concentration range.

**Table 3:** Results of linear relationship examination.

Ingredient	Regression equation	Linear range (mg/mL)	*r*
Caffeic acid	*Y* = 50254*X* – 45.958	0.0096–0.1157	0.9994
Salviaflaside	*Y* = 14802*X* + 18.836	0.00832–0.2080	0.9994
RA	*Y* = 27467*X* – 95.578	0.0148–0.2960	0.9994

Precision evaluation: The RSD of relative retention time for caffeic acid was 0.17%, salviaflaside was 0.37%, RA was 0.24%; the RSD of peak area for caffeic acid was 1.05%, salviaflaside was 1.70%, RA was 0.94%, indicating good precision of the instrument.

Stability assessment: The RSD of caffeic acid peak area was 1.34%, salviaflaside peak area was 2.01%, and RA peak area was 0.83%, indicating good stability of solution within 24 h.

Repeatability assessment: The average content of caffeic acid was 5.82 mg/g with an RSD of 1.32%; the average content of salviaflaside was 23.59 mg/g with an RSD of 2.20%; and the average content of RA is 99.92 mg/g with an RSD of 0.72%, indicating good method repeatability.

Recovery rate test: The average recovery rate of caffeic acid was 95.83%, with an RSD of 1.94%; the average recovery rate of salviaflaside was 99.2%, with an RSD of 1.29%; the average recovery rate of RA was 101.52%, with an RSD of 1.26%, indicating good method accuracy.

Sample content determination: The average content of caffeic acid in the sample was 5.84 ± 0.08 mg/g, the average content of salviaflaside was 23.39 ± 0.24 mg/g, and the average content of RA was 100.43 ± 0.35 mg/g. RA was identified as the predominant component in the PVP extract.

### Inhibition of PVP or RA on CEase activity

#### The inhibitory effect of PVP on CEase

**Figure 2:**
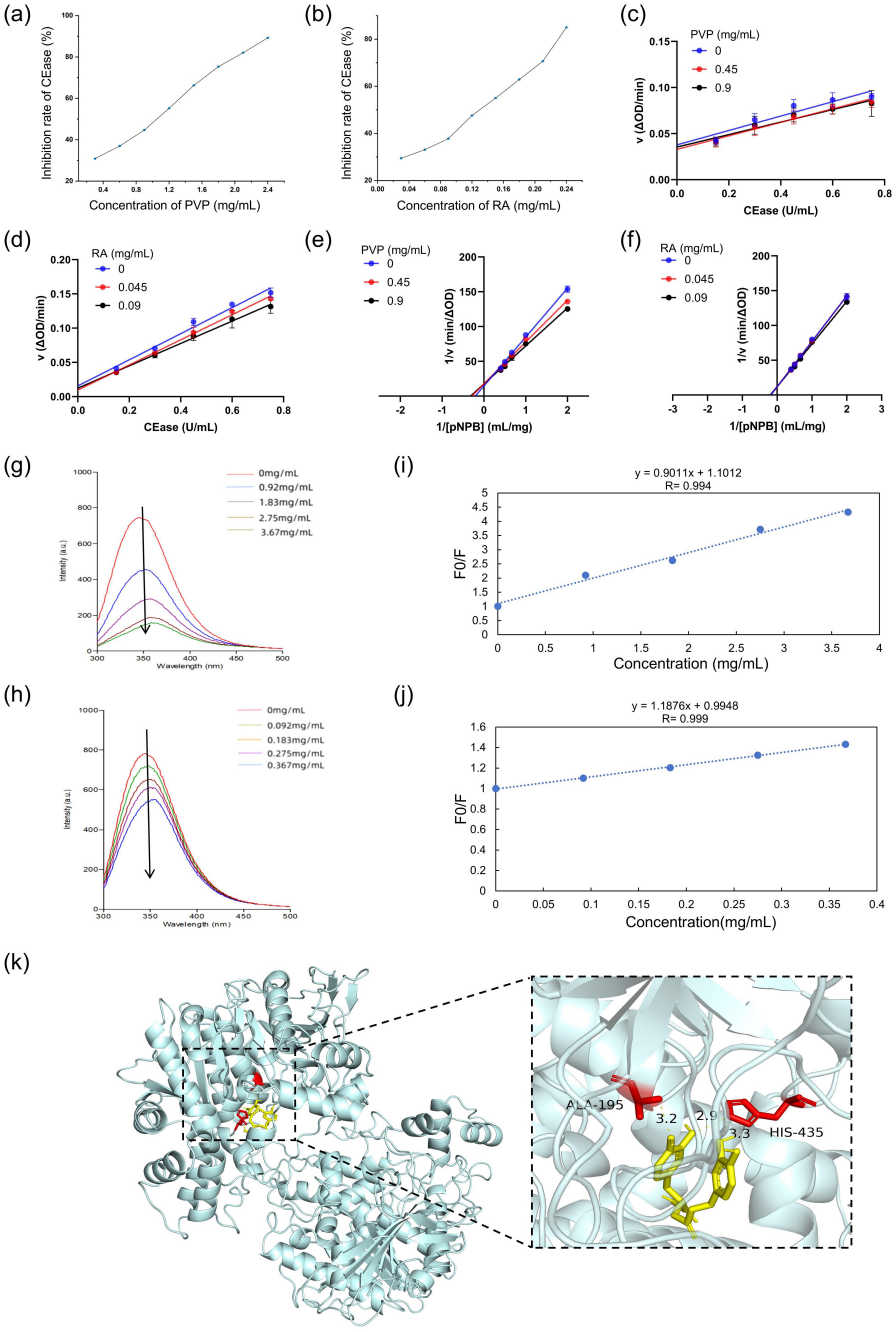
Inhibition of CEase by PVP **(a)** or RA **(b)** at the various concentrations (mg/mL). Kinetic studies of CEase inhibition by PVP **(c, e)** or RA **(d, f)** plotted by Dixon plot and Lineweaver–Burk plot. Fluorescence quenching and interaction mechanism between CEase and PVP **(g, i)** or RA **(h, j)**. **(k)** Molecular docking results of RA with CEase.

As shown in Figures 2a, b, the inhibitory effects of PVP and RA on CEase activity increased with higher concentrations of PVP. The IC_50_ of PVP for CEase inhibition was 1.63 ± 0.06 mg/mL, while that of RA was 0.39 ± 0.17 mg/mL. These results indicate that both PVP and its primary chemical component, RA, exhibit significant inhibitory effects on CEase.

#### Inhibition types of CEase by PVP

As shown in Figures 2c, d, when the enzymatic reaction rate (*v*, ΔOD/min) was plotted against CEase concentration (0.15, 0.3, 0.45, 0.6, and 0.75 U/mL), all lines intersected the coordinate axes. Moreover, the slopes of the lines gradually decreased with increasing concentrations of PVP or RA, indicating that the inhibition process of CEase by PVP or RA is reversible. This suggests the occurrence of non-covalent intermolecular interactions between PVP or RA and CEase, such as electrostatic forces, van der Waals forces, hydrophobic interactions, and hydrogen bonding forces.

Further analysis of the inhibition type of PVP or RA on CEase was conducted using the Lineweaver-Burk double-reciprocal plot method. The results (Figures 2e, f) showed that the plots of PVP or RA intersected with CEase in the first quadrant, with the *K*_*m*_ value decreasing significantly as the concentration increased, while the *V*_*max*_ value decreased with increasing concentration. This indicates that the inhibition mode of PVP or RA on CEase is mixed competitive (Alves et al., 2025). PVP or RA can bind to the CEase-pNPB complex to form an inactive ESI complex, thereby achieving an inhibitory effect.

#### The influence of PVP and RA on the fluorescence spectra of CEase

The interaction and binding affinity between protein fluorophores and ligands can be investigated through fluorescence quenching. We detected the quenching of CEase fluorophores by PVP and RA at an excitation wavelength of 280 nm. As shown in Figure 2g, PVP exhibited a significant quenching effect on CEase, and the fluorescence intensity of CEase decreased with increasing PVP concentration, while the characteristic peak gradually broadened. A notable red shift was observed in the maximum emission wavelength of CEase. After the addition of PVP, the maximum fluorescence emission wavelength of CEase changed from 346.00 to 361.07 nm. This indicates that PVP alters the microenvironment around aromatic amino acids, leading to increased polarity and reduced hydrophobicity. Therefore, the combination of PVP and CEase alters the molecular conformation of CEase, leading to reduced enzymatic activity. According to the Stern-Volmer plot (Figure 2i and Table 4), the binding site number (*n*) is close to 1, indicating at least one optimal binding site between PVP and CEase. Next, we examined the quenching effect of RA on the CEase fluorophore at an excitation wavelength of 280 nm. As shown in Figure 2h, after the addition of RA, the maximum fluorescence emission wavelength of CEase changed from 346.07 to 364.00 nm. The Stern-Volmer plot (Figure 2j) shows that the binding site number (*n*) is close to 1, suggesting at least one optimal binding site between RA and CEase as well.

**Table 4:** *Ksv, K*α, and *n* values of PVP and RA binding to Cease.

Ligands	*Ksv* (mL/mg)	*Kα* (mL/mg)	*n*
PVP	1.00 ± 0.14	0.89 ± 0.05	1.18
RA	1.15 ± 0.23	0.85 ± 0.16	0.90

#### Binding mode of RA- CEase complex

The interaction mode between RA and CEase was analyzed through molecular docking. As shown in Figure 2k, the binding energy between RA and CEase at the site is −8.5 kcal/mol. Combined with the three-dimensional pattern diagram, RA interacts with ALA-195 and HIS-435 at or outside the active site, forming three hydrogen bonds with bond lengths of 3.2Å, 2.9Å, and 3.3Å, respectively, indicating that RA can form stable binding with CEase.

### The effect of PVP on lipid accumulation in HepG2 cells induced by FFA

**Figure 3:**
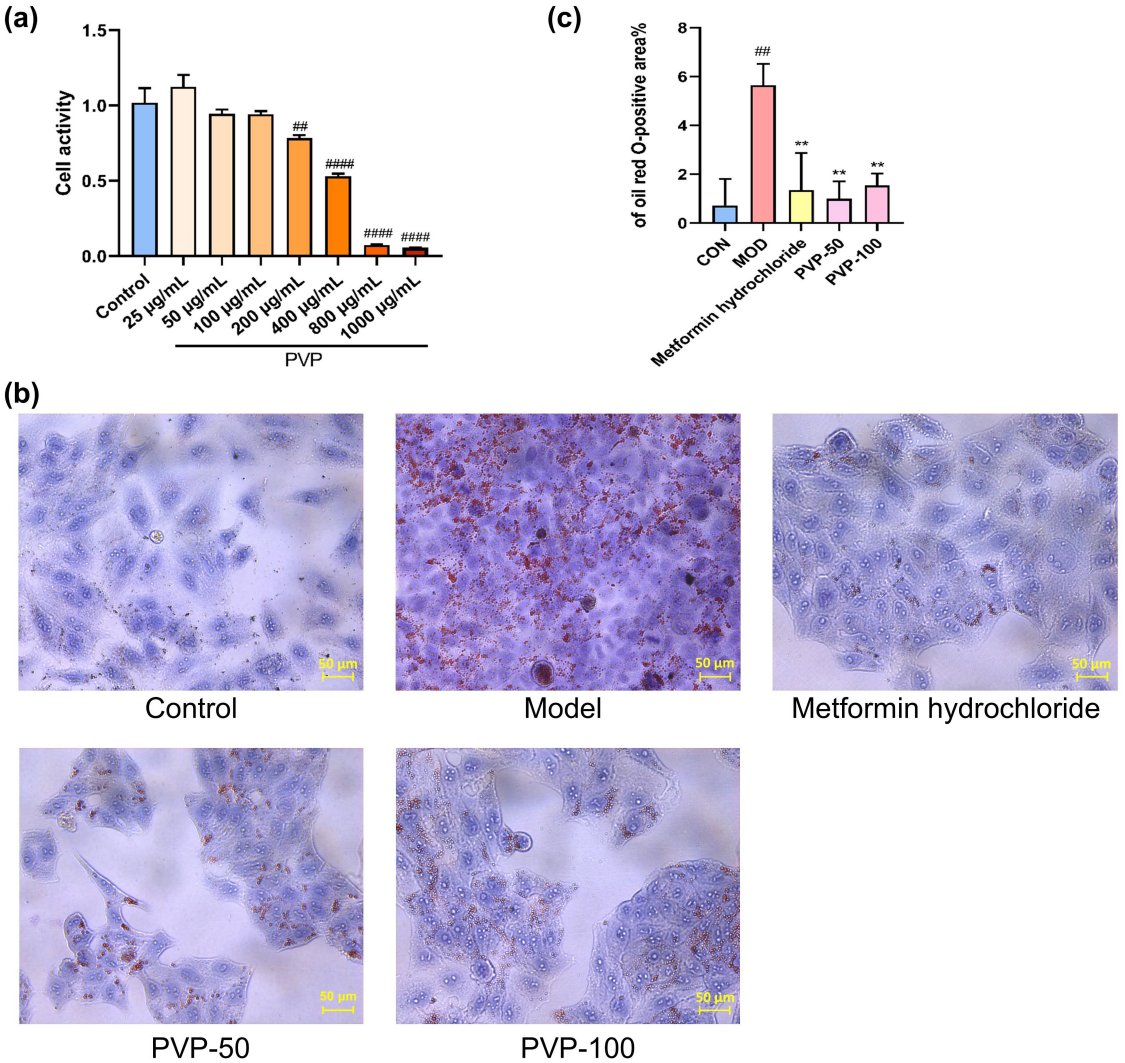
Effects of PVP on FFA-induced lipid accumulation in HepG2 cells. **(a)** The cell viability of HepG2 cells with different concentrations of PVP. **(b)** PVP intervention on lipid accumulation in HepG2 cells after 24 h of FFA induction (Oil Red O staining, 200 × ). **(c)** Percentage of Oil Red O positive area. Analyze the differences between different treatments through one-way ANOVA and Tukey test to form statistical groups. Compared with the control group, ^##^*P* < 0.01, ^####^*P* < 0.0001. Compared with the model group, **P < 0.01.

HepG2 cells, previously induced with FFA for 24 h, were treated with PVP at concentrations ranging from 25 to 1,000 μg/mL. The effect of PVP on cell viability was assessed using the CCK-8 assay. As shown in Figure 3a, compared with the control group, PVP at concentrations of 25–100 μg/mL did not exhibit a significant impact on cell viability. Therefore, subsequent Oil Red O staining experiments were conducted using PVP at 50 and 100 μg/mL.

Oil Red O staining demonstrated (Figure 3b) that compared with the control group, tissue sections in the model group exhibited red lipid staining by Oil Red O, indicating severe lipid droplet accumulation (*p* < 0.01). In contrast to the model group, a significant reduction in lipid droplets were showed in the Metformin hydrochloride, PVP-50, and PVP-100 groups (Figure 3c; *p* < 0.01).

### Inhibited cholesterol transport from Caco-2 cells monolayer

**Figure 4:**
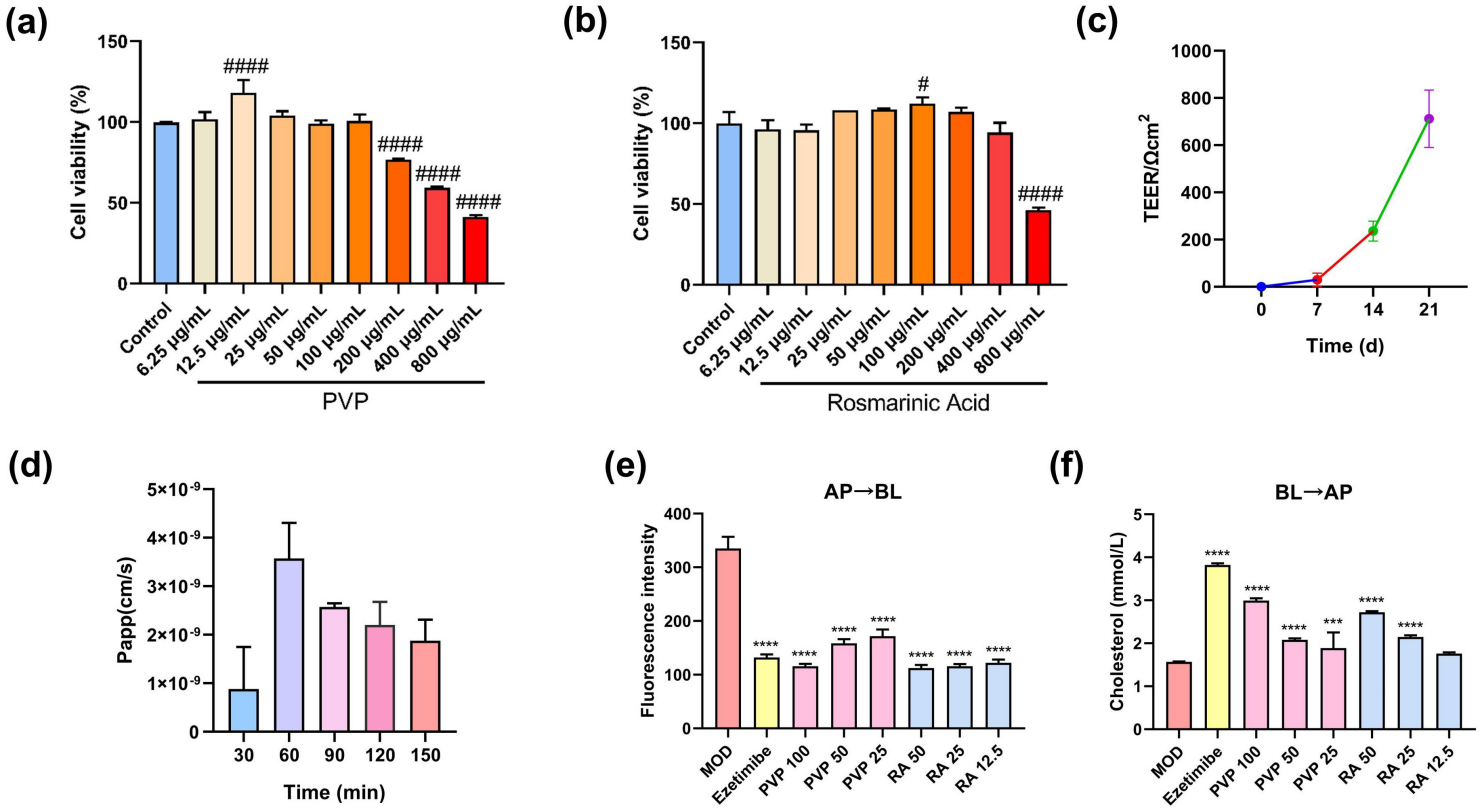
Effects of PVP on cholesterol transport in Caco-2 cell monolayers. **(a, b)** The effects of different concentrations of PVP and RA on the cell viability of Caco-2 cells. **(c)** The resistance value of Caco-2 cells differentiated on transwell. **(d)** Papp. **(e)** Intestinal absorption of cholesterol. **(f)** TICE measurement. Analyze the differences between different treatments through one-way ANOVA and Tukey test to form statistical groups. Compared with the control group, ^#^*P* < 0.05, ^####^*P* < 0.0001. Compared with the model group, ***P < 0.001, and ****P < 0.0001.

Caco-2 cells were treated with PVP or RA at concentrations ranging from 6.25 to 800 μg/mL, and the effects of PVP or RA on cell viability were assessed using the CCK-8 assay. The results (Figures 4a, b) showed that, compared with the control group, PVP at 6.25~100 μg/mL and RA at 6.25~400 μg/mL had no significant effect on Caco-2 cell viability. Therefore, subsequent cholesterol efflux and uptake experiments were conducted using PVP at 25, 50, and 100 μg/mL, and RA at 12.5, 25, and 50 μg/mL.

The TEER of Caco-2 cell monolayers reflects the tightness and integrity of the cell layer. A TEER value greater than 600 Ω·cm^−2^ indicates that the Caco-2 cells have formed a tight monolayer suitable for experiments. With increasing culture time, the actual TEER value of the cells gradually increased. After 21 days, the measured TEER exceeded 600 Ω·cm^−2^, meeting the experimental requirements (Figure 4c). Lucifer Yellow CH is an organic compound with strong yellow-green fluorescence and low permeability to cell membranes, making it suitable as a tracer for assessing membrane permeability. The permeability of the Caco-2 model was evaluated by measuring the difference in Lucifer Yellow CH concentration between the apical (AP) and basolateral (BL) chambers of the Transwell system. The results indicated that within 30–150 min, the Papp values were all below 1 × 10^−6^ cm/s, confirming the formation of a tight monolayer structure in Caco-2 cells (Figure 4d). Compared with the model (MOD) group, both PVP and RA significantly inhibited cholesterol uptake (Figure 4e) and promoted cholesterol efflux in Caco-2 cells (Figure 4f).

### The effect of PVP on serum liver injury markers and lipid levels in MASLD mice

**Figure 5:**
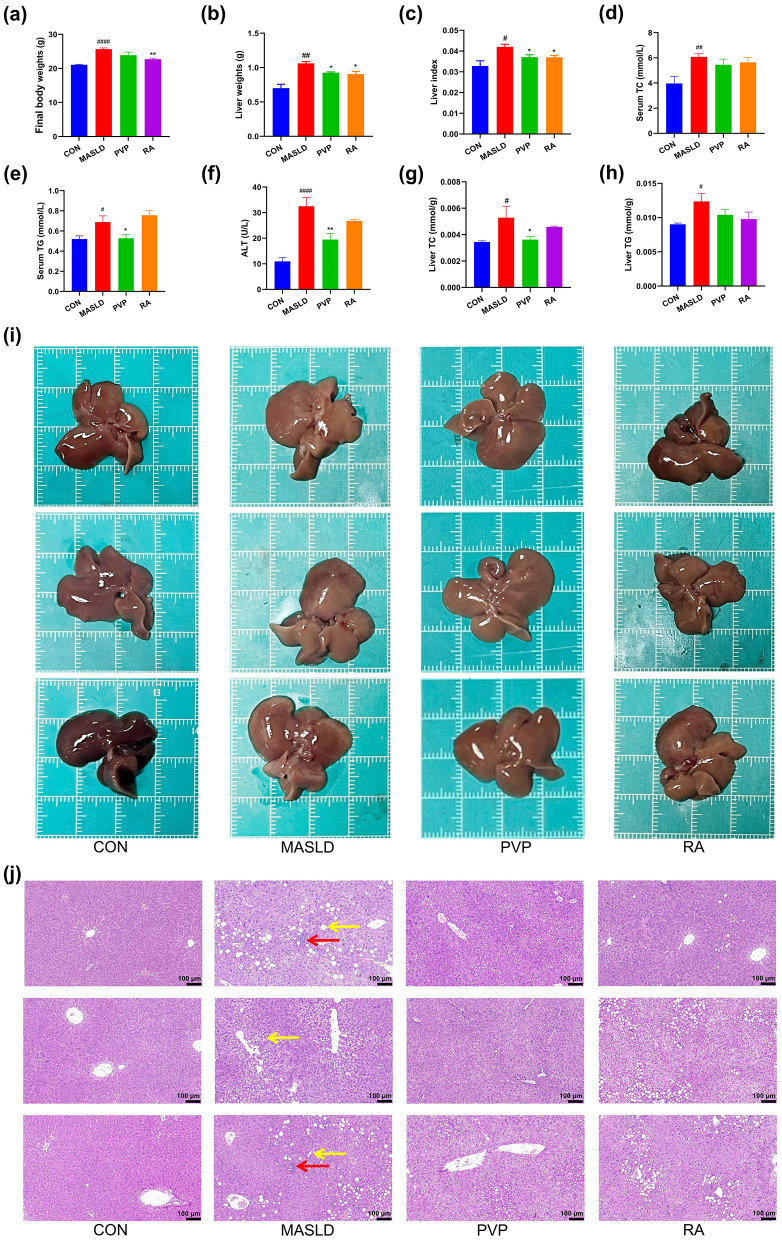
Determination of biochemical indicators and pathological analysis of liver tissue. **(a)** Weight. **(b)** Liver weight. **(c)** Liver coefficient. **(d)** Serum TC level. **(e)** Serum TG level. **(f)** Serum ALT level. **(g)** Liver TC level. **(h)** Liver TG level. **(i)** Mouse liver. **(j)** Liver H & E staining. The red arrow indicates inflammatory infiltration, and the yellow arrow indicates the lipid droplet. Analyze the differences between different treatments through one-way ANOVA and Tukey test to form statistical groups. Compared with the control group, ^#^*P* < 0.05, ^##^*P* < 0.01, ^###^*P* < 0.0001. Compared with the MASLD group, *P < 0.05, **P < 0.01.

Compared with the control group, the MASLD group exhibited a significantly increase in body weight, liver weight, and liver index (*p* < 0.05). In contrast, both PVP and RA effectively attenuated the high-fat choline-deficient diet-induced elevations in body weight, liver weight, and liver index (Figures 5a–c). In addition, serum levels of TC, TG, and ALT were significantly elevated in the MASLD group relative to the control, while PVP significantly reduced TG and ALT levels (Figures 5d–f, *p* < 0.05).

### The effect of PVP on the pathological changes of liver in MASLD mice

As shown in Figures 5g–j, compared with the normal group, the liver color of mice in the MASLD group appeared pale, with abnormal hepatocyte morphology, the presence of numerous vacuoles, and significant immune cell infiltration. Additionally, hepatic TC and TG levels of were significantly increased in the MASLD rats (*p* < 0.05). In contrast to the MASLD group, both the PVP and RA groups exhibited alleviated liver tissue injury, reduced vacuolization in hepatocytes, diminished ballooning degeneration, and decreased levels of TC and TG in the liver.

### The effect of PVP on gut microbiota in MASLD mice

**Figure 6:**
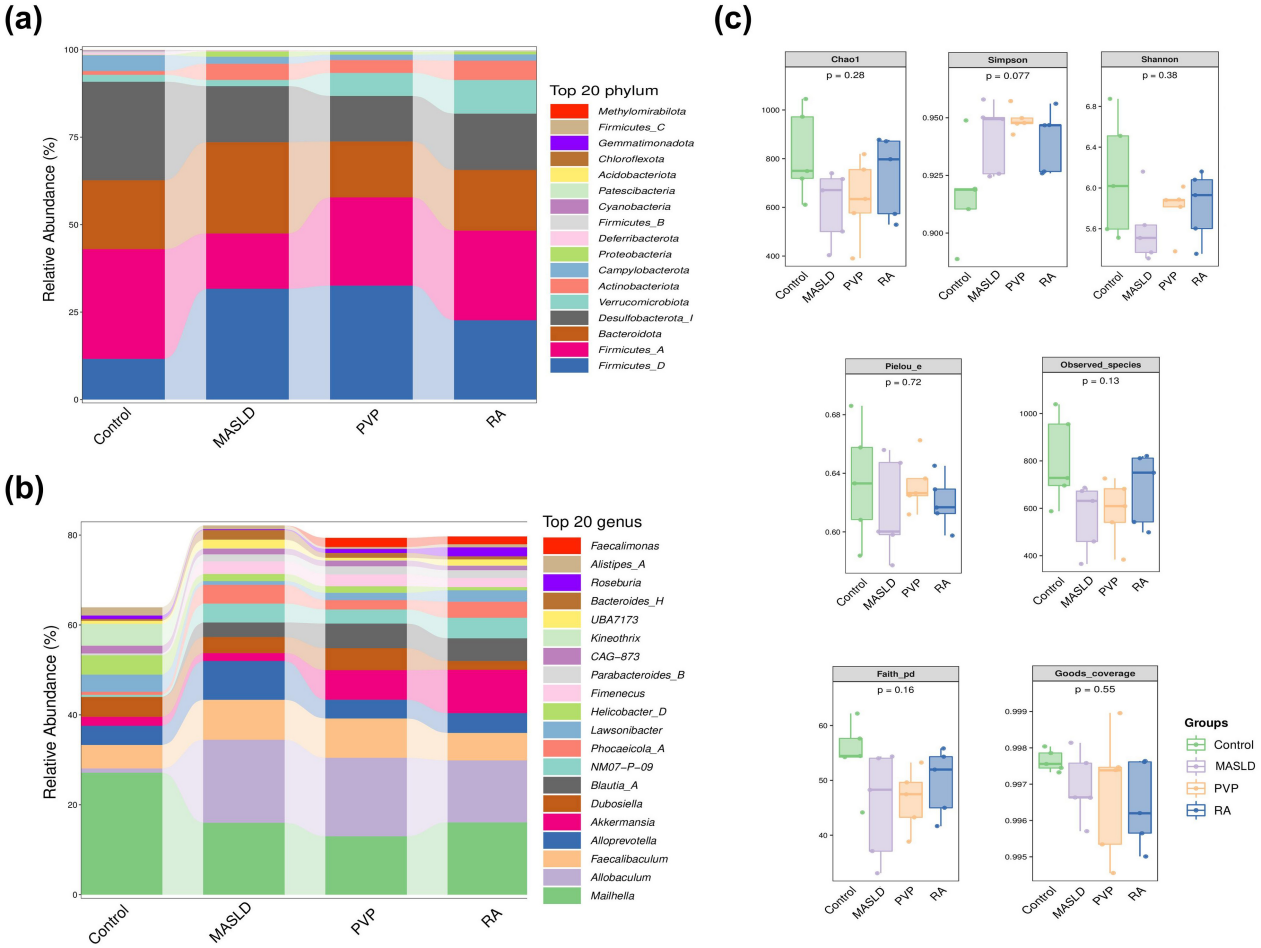
Effects of PVP on the species composition of gut microbiota in MASLD mice. **(a)** Bar chart of species composition at the phylum level. **(b)** Bar chart of species composition at the genus level. **(c)** Alpha Diversity Indices.

The gut microbiota composition of mice in each group was analyzed using 16sRNA sequencing. At the phylum level, *Bacteroidota* and *Firmicutes* are the most abundant phyla (Figure 6a). Compared with the normal group, the abundance of *Bacteroideta* in the model group increased (fold change 1.33), while the abundance of *Firmicutes A* and *Firmicutes B* decreased (fold changes of 0.50 and 0.07, respectively). Compared with the MASLD group, the abundance of *Bacteroidetes* in the PVP group decreased (fold change 0.61), while the abundance of *Firmicutes A* and *Firmicutes B* increased (fold changes of 1.60 and 5.66, respectively). Compared with the MASLD group, the abundance of *Bacteroidetes* in the RA group decreased (fold change 0.66), while the abundance of *Firmicutes A* and *Firmicutes B* increased (fold changes 1.62 and 6.94, respectively). Overall, the *Firmicutes*-all (i.e., the combination of *Firmicutes-A, Firmicutes-B, Firmicutes-C*, and *Firmicutes-D*) and *Bacteroidota* ratios (F/B ratio) in the normal group, MASLD group, PVP group, and RA group were 2.21, 1.82, 3.63, and 2.80, respectively, indicating that MASLD led to a reduction in the gut microbiota F/B ratio in mice, while both PVP and RA interventions restored the F/B ratio. At the genus level (Figure 6b), the MASLD group showed increased abundances of *Alloprevotella, Bacteroides_H, Phocaeicola_A, UBA7173, Dorea_A, Allobaculum, Faecalibaculum, Turicimonas*, and *Escherichia*, and decreased abundances of *Roseburia, Lawsonibacter, Angelakisella, Formimonas*, and *Akkermansia* compared with the normal group. Both PVP and RA interventions reversed these alterations in abundance of these genera.

Compared to the normal group, the MASLD group exhibited a decrease in the Shannon and Pietrou-e indices of the gut microbiota, while PVP and RA increased the above indices (Figure 6c), indicating that the ameliorative effect of PVP on MASLD is associated with the restoration of gut microbiota species composition and diversity.

**Figure 7:**
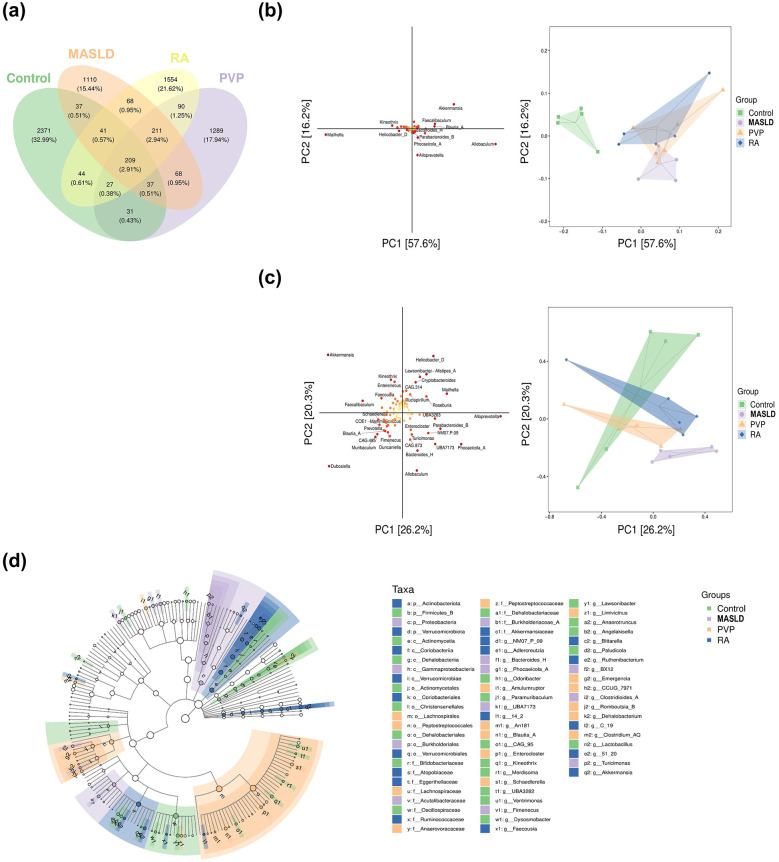
Species differences analysis. **(a)** Species difference analysis and ASV/OTU Venn diagram of marker species. **(b)** Species difference analysis and PCA analysis of marker species. **(c)** Species difference analysis and OPLS-DA analysis of marker species. **(d)** LEfSe analysis.

Figure 7a visually displays the distribution of gene fragments detected in the four experimental groups via a Venn diagram. Among the 2797 gene fragments in the normal group, 11.58% were not detected in the MASLD group, further revealing alterations in the gut microbiota of MASLD subjects. In addition, 70.52% of the gene fragments in the MASLD group were completely absent in the PVP group, and 76.43% were completely absent in the RA group, indicating that both PVP and RA exert significant regulatory effects on the gut microbiota. It is worth noting that 1379 gene fragments in the PVP group were undetected in both the normal and MASLD groups, while 1644 gene fragments in the RA group were undetected in both the normal and MASLD groups.

Species differences analysis and PCA as well as OPLS-DA of biomarker species both indicated distinct clustering of sample points among groups, demonstrating intergroup differences. *Akkermansia, Helicobacter-D, AlloPrevotella*, and *Allobaculum* were identified as the species contributing most significantly to compositional differences between sample groups (Figures 7b, c). Figure 7d shows the significantly enriched species in each group along with their importance levels. *Acutalibacteraceae* and *Phocaeicola-A* were the most abundant differential biomarker species at the family and genus levels in the MASLD group, respectively. *Akkermansiaceae* and *Akkermansia* were the most abundant differential biomarker species at the family and genus levels in the RA group, respectively. *Lachnospiraceae* and *Blautia_A* were the most abundant differential biomarker species at the family and genus levels in the PVP group, respectively.

### The effect of PVP on BAs in MASLD mice

**Figure 8:**
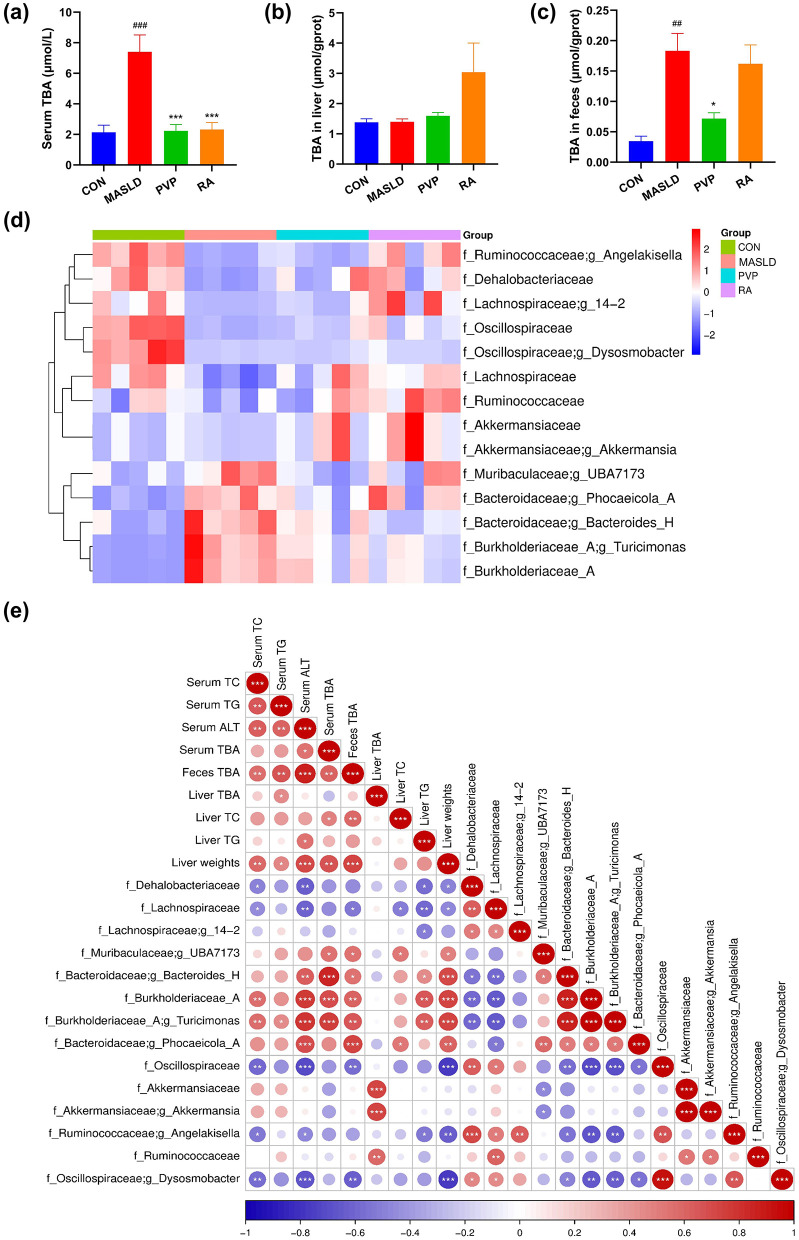
The effect of PVP on BA levels in MASLD mice. **(a)** Serum TBA levels. **(b)** Liver TBA levels. **(c)** Fecal TBA levels. **(d)** Heatmap of the abundance of major differential species. **(e)** Correlation coefficient matrix plot. Analyze the differences between different treatments through one-way ANOVA and Tukey test to form statistical groups. Compared with the normal group, ^##^*p* < 0.01, ^###^*p* < 0.001. Compared with the MASLD group, *p < 0.05, **p < 0.01, and ***p < 0.001.

Compared to the normal group, the MASLD group exhibited significantly elevated serum TBA levels and fecal TBA levels (*p* < 0.01), while no significant difference was observed in liver TBA level. In comparison with the MASLD group, both the PVP and RA groups showed significantly reduced serum TBA levels, and the PVP group demonstrated significantly decreased fecal TBA levels (*p* < 0.05), with no significant differences in liver TBA levels (Figures 8a–c). In this experiment, a total of 14 differential bacteria showed opposite trends in relative abundance between the MASLD vs. CON comparison and either the PVP vs. MASLD or RA vs. MASLD comparisons (Figure 8d). Subsequently, Pearson correlation analysis was performed between the abundance of these 14 major differential bacteria and biochemical indicators. The results showed (Figure 8e) that fecal TBA, serum TC, *UBA7173, Bacteroides_H, Burkholderiaceae_A*, and *Turicimonas* were significantly positively correlated with serum TBA levels (*p* < 0.05). Serum TC, *UBA7173, Bacteroides_H, Burkholderiaceae_A, Turicimonas*, and *Phocaeicola_A* were significantly positively correlated with fecal TBA levels (*p* < 0.05). In contrast, *Lachnospiraceae, Oscillospiraceae, Dyssomobacter* were significantly negatively correlated with fecal TBA levels (*p* < 0.05).

## Discussion

In this study, we conducted intervention studies with PVP and RA on MASLD mice induced by an 8 weeks high-cholesterol choline-deficient diet. Our research results demonstrate that PVP and RA ameliorate MASLD through multiple mechanisms, including reductions in body weight, liver weight, serum TG and ALT levels, as well as hepatic TC level in MASLD mice, reducing hepatic lipid accumulation, inhibiting intestinal cholesterol absorption, regulating BA metabolism and associated intestinal bacteria. To our knowledge, this is the first study to evaluate the potential efficacy of PVP in the treatment of MASLD.

Liver is the primary organ responsible for regulating lipid metabolism. The pathogenesis of MASLD was thought to result from lipotoxic injury to hepatocytes caused by accumulated triglycerides. However, recent studies have shown that discrupted cholesterol homeostasis plays a more critical role (Li et al., 2024c). Dietary exogenous cholesterol is absorbed in the intestine and transported to the liver via chylomicrons. Excessive intake of cholesterol leads to cholesterol accumulation in the liver and blood, contributing to pathological conditions such as fatty liver and atherosclerosis (Li et al., 2024c). Research has shown that inhibiting the absorption of cholesterol/phytosterols at the apical brush border membrane of small intestinal epithelial cells can improve high-fat/cholesterol-induced MASLD (Zheng et al., 2008). For example, curcumin has been found to alleviate high-fat diet induced hepatic cholesterol accumulation and steatosis by reducing intestinal cholesterol absorption and hepatic biliary cholesterol reabsorption (Yang et al., 2023). Polyphenol extracts from *Prunus cerasifera* Ehrhart alleviate MASLD by reducing total hepatic cholesterol, increasing hepatic TBAs, inhibiting intestinal cholesterol absorption, and enhancing intestinal cholesterol efflux (Ren et al., 2024). CEase accelerates intestinal cholesterol absorption by hydrolyzing phosphatidylcholine (PC). Various plant polyphenols have demonstrated significant inhibitory effects on CEase (He et al., 2023). In this study, both PVP and RA were found to inhibit CEase activity in a mixed competitive manner. This suggests that the ameliorative effects of PVP and RA on MASLD may be associated with the inhibition of intestinal cholesterol absorption.

Multiple studies have shown that polyphenols have the potential to reverse or enhance features associated with MASLD by affecting gut microbiota and regulating the gut-liver axis (Li et al., 2025). MASLD patients have a higher abundance of Gram-negative bacteria in the intestines, with a 20% increase in *Bacteroidetes* and a 24% decrease in *Firmicutes* (Safari and Gerard, 2019; Wang et al., 2016). At the familial level, the most prevalent bacteria in MASLD patients are *Bacteroideceae* and *Enterobacteriaceae* (Jin et al., 2024). Phenolic acids can be absorbed through the small intestine, large intestine, and colon, thereby regulating and controlling the activity of various enzymes associated with diseases and inflammation (Li et al., 2025). In addition, phenolic acids can regulate gut microbiota, for example, polyphenols increase the proportion of butyrate-producing bacteria such as *Blautia* and *Dorea* within the family *Lachaospiraceae*, while suppressing the growth of *Bacteroides* and *Desulfovibrio brassicaceae* associated with disease and inflammation (Yang et al., 2019). Caffeic acid prevents high-fat diet-induced MASLD by reversing gut microbiota imbalance and associated lipopolysaccharide-mediated inflammation (Mu et al., 2021). In the present study, both PVP and RA increased the abundance of *Firmicutes* and decreased that of *Bacteroidetes*, restoring the elevated levels of *Bacteroidaceae* and *Enterobacteriaceae*, as well as the reduced level of *Akkermansia*, in the cecum of MASLD mice. Previous studies have shown that the abundance of the *Bacteroidaceae* family and *Bacteroides* genus is associated with the alleviation of early-stage NASH in a Western diet (WD)/CCl_4_-induced mouse model (Li et al., 2024b). *Akkermansia muciniphila* can improve MASLD by reshaping gut microbiota, regulating BA metabolism, and modulating FXR expression (Juárez-Fernández et al., 2021; Nian et al., 2023).

The imbalance of gut microbiota is closely related to the onset and progression of MASLD through the action of metabolites such as BAs, lipopolysaccharides, choline, and short chain fatty acids (Mohammadhasani et al., 2024). Among them, BAs are amphiphilic molecules that are first synthesized from cholesterol in liver cells and then metabolized by gut microbiota. Imbalance of gut microbiota can disrupt the synthesis and conversion of BAs, thereby affecting the digestion and absorption of lipids in the body. In this study, we found that the levels of TBAs in the feces and serum of MASLD mice were significantly increased, which is consistent with literature reports (Adams et al., 2020). The increase in fecal BAs is driven by secondary non-conjugated BAs (mainly DCA), and the level of DCA in feces is correlated with the abundance of *Bacteroidaceae* and *Lachnospiraceae* (Adams et al., 2020). In this study, *Lachnospiraceae, Oscillospiraceae*, and *Dyssomobacter* were significantly negatively correlated with fecal TBA levels. Certain BA 7α-dehydroxylating bacteria associated with the family *Osillospiraceae* are primarily responsible for converting primary BA into secondary BAs in the human gut (Kim et al., 2022). In addition, *Angelakisella* belongs to a probiotic (Wang et al., 2024b), whose relative abundance decreased in the fecal microbiota of high-fat diet-fed mice (Daniel et al., 2021), and may be involved in BA metabolism (Plaza-Díaz et al., 2022). This study observed a significant reduction in the abundance of *Angelakisella* in the cecum of MASLD mice, along with a certain negative correlation with serum and fecal TBA levels. After intervention with PVP and RA, its abundance increased. This suggests that PVP and RA may promote the reabsorption of secondary BAs by modulating the gut microbiota, thereby ameliorating MASLD.

However, there are several critical translational challenges must be acknowledged before any clinical or real-world application can be realistically considered. First, the bioavailability and pharmacokinetics of PVP and RA are yet to be determined. Future pharmacokinetic and toxicological studies will be needed to refine these estimates and assess safety in humans. Second, the specific microbial shifts observed in C57BL/6 mice may not generalize to humans with different baseline microbiota. The efficacy of PVP and RA could be population-dependent or even person-dependent. Third, there are well-recognized differences between mouse MASLD models and human MASLD. Human MASLD is often associated with multiple comorbidities (type 2 diabetes, hypertension, dyslipidemia) and develops over decades, whereas our model represents a relatively acute or subchronic intervention. Fourth, shotgun metagenomics and targeted bile acid metabolomics studies should be conducted on cecal contents in future work to establish causal relationships and supported the claim that PVP regulates “BA metabolism.” Fifth, The CD-HFD model has limitations, including the absence of choline, which affects phospholipid and one-carbon metabolism, and its mechanisms do not fully replicate those in human MASLD/MASH patients. The future studies using more clinically relevant models (e.g., long-term HFD or Gubra-Amylin NASH diet) are warranted. In summary, our findings provide a mechanistic basis for the beneficial effects of PVP and RA on MASLD.

**Figure 9:**
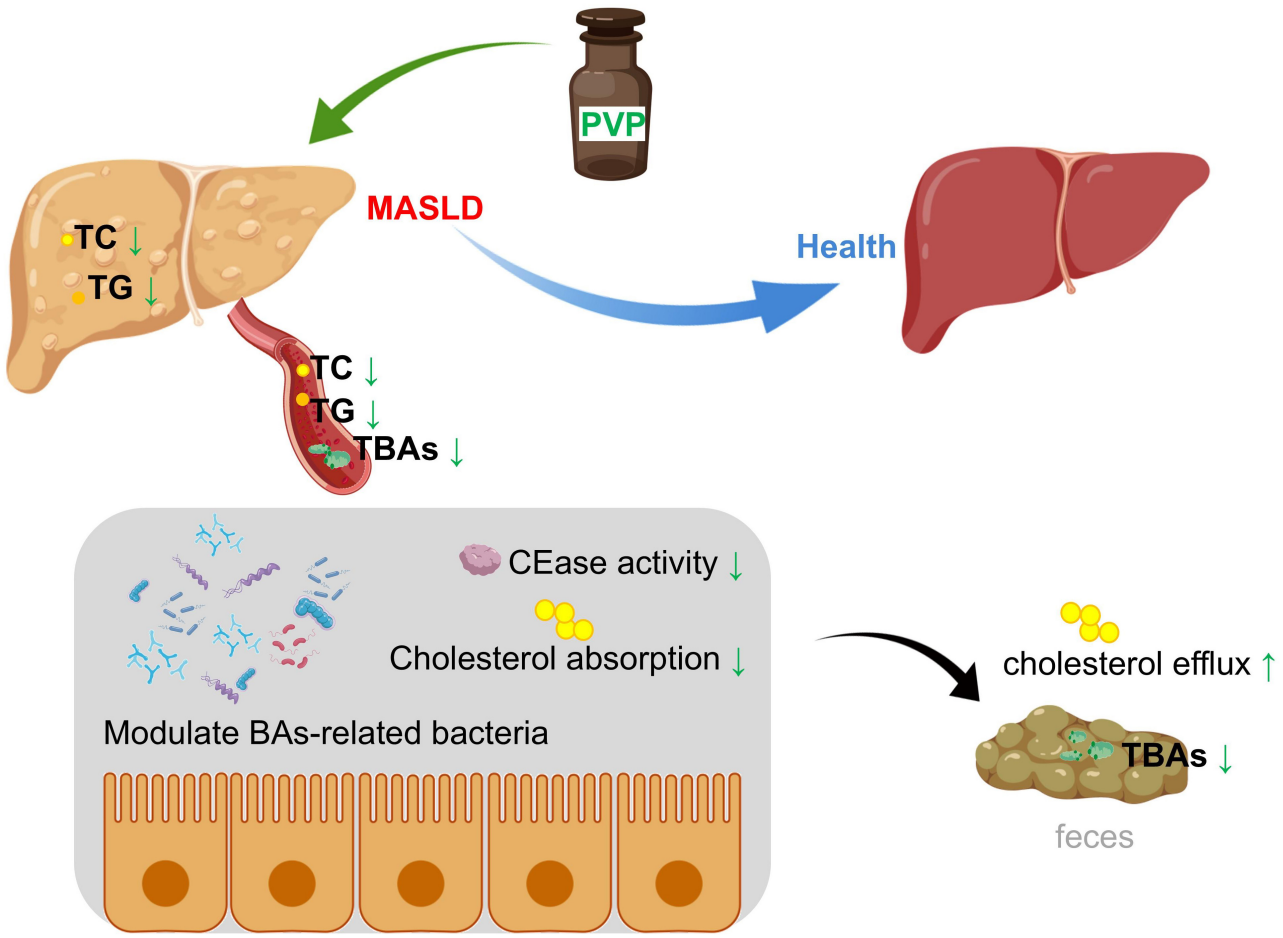
The mechanism diagram of PVP mediate the gut-liver axis to improve MASLD.

In summary (Figure 9), this study demonstrates for the first time that PVP and RA ameliorate MASLD through multiple mechanisms, including reductions in body weight, liver weight, serum TG, ALT, and TBA levels, hepatic TC content, and fecal TBA levels in MASLD mice, along with attenuated hepatic steatosis, inhibited CEase activity and intestinal cholesterol absorption, and promoted cholesterol efflux. Additionally, PVP and RA modulate TBA levels by upregulating the abundance of BA-related bacteria such as *Lachnospiraceae* and *Oscillospiraceae*, while downregulating *UBA7173, Bacteroides_H, Burkholderiaceae_A, Turicimonas*, and *Phocaeicola_A*. These findings provide new insights into the application of natural products in the development of MASLD therapeutics.

## Data Availability

The raw data generated in this study can be found in the China National Centre for Bioinformatics (https://www.cncb.ac.cn) under project no. PRJCA065959.
